# Severe isolated thrombocytopenia after clopidogrel and pentoxifylline therapy: a case report

**DOI:** 10.1186/1752-1947-5-281

**Published:** 2011-07-04

**Authors:** Elisa Celeste da Silva Vedes, Lia Dulce Guerreiro Marques, Miguel Cordovil Toscano Rico

**Affiliations:** 1 Departamento de Medicina, Centro Hospitalar Lisboa Norte, Hospital Pulido Valente, Lisboa, Portugal; 2 Departamento de Medicina, Centro Hospitalar Lisboa Central, Hospital de Santa Marta, Lisboa, Portugal

## Abstract

**Introduction:**

Clopidogrel is frequently associated with thrombotic thrombocytopenic purpura, however this drug is rarely related to severe isolated thrombocytopenia. Pentoxifylline has previously been associated with thrombocytopenia only once. To the best of our knowledge, this is the first report of severe isolated thrombocytopenia after therapy with both clopidogrel and pentoxyfilline.

**Case presentation:**

We report the case of a 79-year-old Caucasian man who presented to our facility with intermittent claudication. He had obliterative arterial disease and started therapy with clopidogrel and pentoxifylline. His basal platelet count was 194 × 10^9 ^cells/L. At three days after the start of treatment, our patient had lower limb petechia and stopped taking clopidogrel and pentoxifylline. His platelet count lowered to 4 × 10^9 ^cells/L and our patient was admitted to hospital. Our patient had purpura with no other hemorrhages or splenomegaly. Results of a blood smear were normal, and a bone marrow study showed dysmegakaryopoiesis. Antiplatelet antibody test results were negative, as were all viral serology tests. Imaging study results were normal. Our patient was given immunoglobulin but there was no sustained platelet increase, so corticotherapy was started as the next treatment step. At five months after clopidogrel and pentoxifylline were discontinued, his platelet count continued increasing even after prednisolone was tapered.

**Conclusions:**

Severe isolated thrombocytopenia may appear as a side effect when using clopidogrel and pentoxifylline. These drugs are widely used by general physicians, internists, cardiologists and vascular surgeons. We hope this report will raise awareness of the need to monitor the platelet count in patients taking these drugs.

## Introduction

Antithrombotic therapy-related thrombocytopenia has been extensively described concerning heparin and ticlopidine therapy. Clopidogrel, as ticlopidine, is a thienopyridine derivative and it is more effective and safer than aspirin in reducing adverse cardiovascular events in patients with atherosclerosis [1]. Clopidogrel acts by inhibiting ADP-induced platelet aggregation and, because of its efficacy, safety profile and tolerability, it is widely used by the medical community. It has been associated with thrombotic thrombocytopenic purpura (TTP) [2]. However, to the best of our knowledge only three reports have linked this drug with severe isolated thrombocytopenia [3-5] and the exact mechanism of hematological dyscrasia associated with clopidogrel remains unclear. Pentoxifylline has been used to relieve intermittent claudication. The precise mode of action of pentoxifylline and the sequence of events leading to clinical improvement are still to be determined, but some consider it to be a hemorheological agent. Pentoxifylline and its metabolites may improve blood flow by increasing red blood cell deformability and decreasing blood viscosity, also reducing platelets aggregation [6]. To the best of our knowledge, there is only one report of pentoxifylline-associated thrombocytopenia [7].

We report a case of clopidogrel plus pentoxifylline associated severe isolated thrombocytopenia.

## Case presentation

Our patient was a 79-year-old Caucasian man with a medical history of hypertension and type 2 diabetes, controlled with candesartan (16 mg/day) and diet. About three weeks before admission to our facility, he visited his general practitioner complaining of intermittent claudication. A lower limb Doppler ultrasound study revealed occluding disease of the left femoral and popliteal sector, with low amplitude flow in the posterior tibial and peroneal arteries. The study also showed disease of the lower genicular sector with low dorsalis pedis flow. Clopidogrel (75 mg/day) and pentoxifylline (400 mg/day) were started due to the obliterative arterial disease, and our patient was referred to a vascular surgeon. He had a normal baseline platelet count of 194 × 10^9 ^cells/L. On the third day after beginning these drugs, our patient reported lower limb petechia and stopped taking them. He had no major bleeding loss. At this time his platelet count was 147 × 10^9 ^cells/L. Our patient attended a vascular consult for the first time, and the vascular surgeon requested another platelet count. On the 17th day, the result was 4 × 10^9 ^platelets/L. Pseudothrombocytopenia was excluded after a peripheral blood smear was performed and our patient was admitted to our internal medicine ward.

On admission, he had purpura in the lower limbs. His blood pressure was 170/85 mmHg, heart rate was 60 beats per minute and respiratory rate was 16 breaths per minute. Consciousness was clear and no neurological abnormality was noted. Our patient had no jaundice or cyanosis. Cardiac and pulmonary observation showed no abnormalities and he did not have abdominal hepatomegaly or splenomegaly (checked with ultrasound).

Severe isolated thrombocytopenia was confirmed (5 × 10^9 ^cells/L), without schistocytes or other abnormalities. His fibrinogen level was normal, as were his haptoglobin and complement levels. Antiplatelet antibody test results were negative. β2-Microglobulin and prostate specific antigen levels were also within normal ranges. There was no evidence of recent viral infection. Viral serology test results, including HIV, were negative. Thoracic, abdominal and pelvic computed tomography scan results were normal. A bone marrow study was performed showing megakaryocytes within normal and dysmegakaryopoiesis.

**Figure 1:**
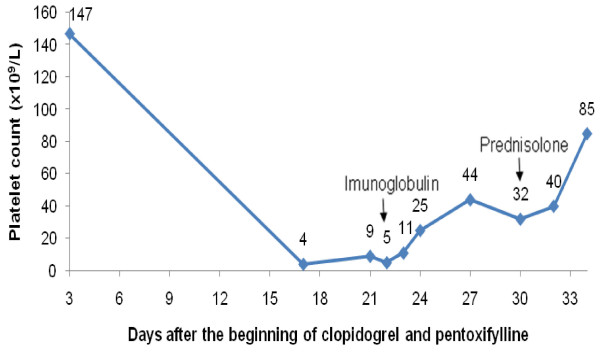
**Platelet count after the beginning of anti-thrombotic therapy with clopidogrel and pentoxifylline**. The arrows mark the dates when immunoglobulin and corticotherapy were started.

**Figure 2:**
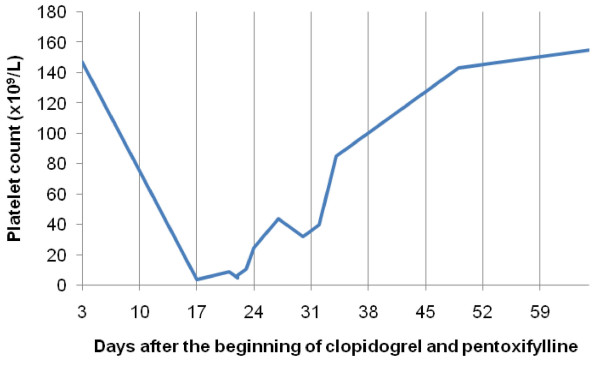
**Platelet count including follow-up after hospital discharge**.

Although clopidogrel and pentoxifylline had been stopped, our patient had 5 × 10^9 ^platelets/L on hospital admission (22nd day) and intravenous immunoglobulin (IgG) was started (0.4 g/kg/day for two days). His platelet count increased to 44 × 10^9 ^platelets/L at five days after admission (27th day after starting clopidogrel and pentoxifylline), but it subsequently decreased again to 32 × 10^9 ^platelets/L (30th day). Prednisolone was given (1 mg/kg/day) and four days later (34th day) his platelet count was 85 × 10^9 ^cells/L and our patient was discharged (Figure 1). At one month after clopidogrel and pentoxifylline were discontinued, platelet count continued to increase (155 × 10^9 ^cells/L with 0.25 mg prednisolone/kg/day) (Figure 2).

Prednisolone was tapered over four months and our patient's platelet count returned to normal levels.

During his stay at the hospital, our patient's blood pressure and glycemia were controlled with an adequate diet with no need for medication. Our patient's claudication remains stable and he continues peripheral artery disease follow-up with a vascular surgeon. Our patient is currently on exercise therapy and our vascular surgery consultant is currently planning to start therapy with aspirin (100 mg/day) under close surveillance. Our patient was not indicated for vascular surgery.

## Discussion

There are several possible etiologies for thrombocytopenia. Firstly, when a low platelet count is obtained, pseudothrombocytopenia must be excluded. Our patient presented with petechia, ruling out this option. Secondly, real thrombocytopenia can be inherited or acquired. Our patient is a 79-year-old man with previous normal platelet count, suggesting an acquired form of thrombocytopenia [8]. Thirdly, acquired thrombocytopenia can be divided in immune and nonimmune causes. We used antiplatelet antibodies as diagnostic adjuvant. However, this test lacks sensibility and interlaboratory reproducibility. Some studies document positive antiplatelet antibody tests in 10% to 20% of patients with certain nonimmune caused thrombocytopenia [9]. Drugs can act as immune cause for thrombocytopenia, through mimicry or as allergens, and induce antiplatelet antibody formation. They can also cause nonimmune thrombocytopenia, suppressing bone marrow thrombopoiesis. Unlike in idiopathic thrombocytopenic purpura, our patient's platelet count did not remain chronically low. Instead it continues rising after corticotherapy tapering, supporting the drug-associated etiology.

After stopping clopidogrel plus pentoxifylline and prescribing intravenous IgG and corticosteroid therapy our patient's platelet count returned to normal. Full recovery was maintained without corticosteroids, confirming drug-related thrombocytopenia.

For patients with peripheral artery occlusive disease and moderate-to-severe disabling intermittent claudication who do not respond to exercise therapy, and who are not candidates for surgical or catheter based intervention, treatment guidelines recommend cilostazol (a type III phosphodiesterase inhibitor that suppresses platelet aggregation and is a direct arterial vasodilator). However, they suggest that clinicians do not use cilostazol in patients with less disabling claudication, as was the case in our patient. For such patients an exercise training program is recommended and antithrombotic therapy may modify the natural history of chronic lower-extremity arterial insufficiency as well as lower the incidence of associated cardiovascular events. Aspirin will delay the progression of established arterial occlusive disease (75 to 325 mg/day) and, in patients without clinically manifest coronary or cerebrovascular disease, it is preferred over clopidogrel. Pentoxifylline may be considered to treat patients with intermittent claudication; however, the anticipated outcome is likely to be of marginal clinical importance. American College of Chest Physicians guidelines recommend against its use [10,11].

## Conclusions

Clopidogrel and pentoxifylline are widely used by general physicians, internists, cardiologists and vascular surgeons. This report raises awareness that severe isolated thrombocytopenia can be a potential side effect in patients medicated with these drugs. The exact mechanism(s) that caused the severe isolated thrombocytopenia remain unclear. In this case we cannot know which drug caused the low platelet count or if it was the association of clopidogrel and pentoxifylline that was responsible for it. No matter which was the case, physicians should be aware that, when using these drugs, there is a possibility that severe thrombocytopenia may appear as a side effect and platelet count must be monitored.

## Consent

Written informed consent was obtained from the patient for publication of this case report and any accompanying images. A copy of the written consent is available for review by the Editor-in-Chief of this journal.

## Competing interests

The authors declare that they have no competing interests.

## Authors' contributions

All authors analyzed and interpreted the patient data regarding the hematological disease. All authors read and approved the final manuscript.
